# Successful Use of Tocilizumab in a Patient with Coexisting Rheumatoid Arthritis and Ulcerative Colitis

**DOI:** 10.1155/2016/7562123

**Published:** 2016-10-12

**Authors:** Matthew Chak Hin Szeto, Metin Devrim Yalçın, Abdul Khan, Andrzej Piotrowicz

**Affiliations:** Queen Elizabeth the Queen Mother Hospital, Margate, UK

## Abstract

Tocilizumab is an interleukin-6 receptor inhibitor licensed for moderate to severe rheumatoid arthritis (RA). We report a case of Tocilizumab monotherapy for severe active RA in a patient with coexisting ulcerative colitis (UC). The patient was intolerant to multiple disease-modifying drugs, so Tocilizumab monotherapy was commenced. We found clinical improvement in both RA and UC. There was no major adverse event after 2 years. Manufacturer advised caution in using Tocilizumab in patient with gastrointestinal ulceration due to an increased risk of bowel perforation. However, alternative treatments such as glucocorticoid and nonsteroidal anti-inflammatory drugs may carry a higher bowel perforation risk. The presence of gastrointestinal ulceration therefore should not constitute an absolute contraindication for Tocilizumab therapy. Future studies of registry data will inform clinician of the Tocilizumab-related risk of gastrointestinal toxicity in “real-life” settings. Contrary to previous case report, we found Tocilizumab therapy to have a positive effect on UC. Laboratory studies supported a role for interleukin-6 in the pathophysiology of UC. Further clinical trial to evaluate the therapeutic role of Tocilizumab in UC would be warranted.

## 1. Introduction

IL6 pathway has been identified as a potential therapeutic target in ulcerative colitis (UC) by animal and in vitro studies [1, 2]. However, no controlled trial to investigate the therapeutic role of Tocilizumab in UC has been conducted.

Tocilizumab, a humanized anti-interleukin-6 (IL6) receptor antibody, is an established treatment for moderate to severe active rheumatoid arthritis (RA) which cannot be adequately controlled by conventional disease-modifying antirheumatic drugs (DMARDs) [3].

The manufacturer cautioned against use of Tocilizumab in patient with coexisting gastrointestinal ulcerations [4], as an increased risk of bowel perforation was reported in clinical trials [5].

We present a case of a 63-year-old female with coexisting RA and UC. After commencing on Tocilizumab monotherapy, she experienced improvement in both UC and RA. No major adverse event was detected after 2 years. This is the first case report of positive clinical response in UC following Tocilizumab therapy.

## 2. Case Report

Our patient presented 10 years ago with bloody diarrhoea. A clinical diagnosis of ulcerative colitis (UC) was made. Subsequent colonoscopy showed left sided proctocolitis. Colonic biopsies demonstrated cryptitis, crypt abscesses, gland distortion, and mucin depletion.

She developed oral and pharyngeal mucositis after being treated with oral Asacol MR tablets. She has declined other oral 5-aminosalicylates preparations for fear of similar adverse reaction. She was taking prednisolone 5 mg once daily for the past 5 years.

She had persistent mild to moderate UC based on Truelove and Witts' severity index. She reported 2-3 loose bowel movements daily with associated faecal urgency. She experienced 2-3 flares per year, which were characterised by increased bowel frequency (up to 10 times a day) and bloody diarrhoea. They usually responded to Asacol suppositories 1 gram twice daily. However, she had a severe flare-up 5 months ago which required additional oral prednisolone to achieve control (40 mg once daily tapering down to 5 mg once daily over 7 weeks).

Flexible sigmoidoscopy showed mucosa erythema and oedema from rectum to splenic flexure. A 3 mm polyp was seen in the distal sigmoid colon. Histological examination of random colonic biopsies showed increase in the lamina propria inflammatory cell population with crypt distortion and foci of cryptitis. The sigmoid polyp showed crypt distortion in keeping with an inflammatory polyp. There was no dysplasia or neoplasia, and no cytomegalovirus inclusion was seen. This was in keeping with active ulcerative colitis.

This patient has coexisting RA, which was diagnosed 6 years before the onset of UC. She was positive for Rheumatoid Factor (52 U/mL) and anticyclic citrullinated protein antibody (219 U/mL). She was intolerant of multiple DMARDs (Table 1).

She was also previously treated with Etanercept (Enbrel) and Infliximab (Remicade) by her rheumatologist.

Etanercept monotherapy was stopped within 2 months due to adverse reactions. Infliximab was given in combination with Methotrexate (initially 5 mg weekly oral dose, subsequently changed to subcutaneous route due to nausea) and was moderately efficacious in treatment of RA. Her Disease Activity Score (DAS28) decreased from 6.27 pre-Infliximab to 4.34 in 6 months.

However, Infliximab was not efficacious in treatment of UC. She continued to suffer from UC flares while on Infliximab, requiring treatment with Asacol suppositories and oral prednisolone. Flexible sigmoidoscopy and biopsies confirmed active proctocolitis despite Infliximab therapy.

Infliximab was discontinued after 11 months due to side effects.

Her RA remained highly active, with DAS28 score of 7.81 (Table 2). She reported morning stiffness lasting 3-4 hours. Blood tests showed elevated Erythrocyte Sedimentation Rate (ESR) of 52 mm/hour. There was microcytic anaemia (Table 3).

She has a background of atrial fibrillation. She was on 5 mg of prednisolone daily, and she was also prescribed regular bisoprolol, co-codamol, and lansoprazole. She is a nonsmoker and does not consume alcohol.

She was commenced on Tocilizumab monotherapy (8 mg/kg every 4 weeks) for RA.

The patient's RA responded well to Tocilizumab, achieving low disease activity by 3 months. Prednisolone was tapered and stopped by 18 months (Table 2). Her laboratory markers of inflammations also improved over the 24-month period (Table 3). Radiographs of her hands and feet showed no structural progression over the 2-year period.

Although Tocilizumab was not prescribed as treatment for UC, the patient reported improvement in her bowel symptoms. She has had no further UC flare and is passing formed stool twice daily.

Her 10-year surveillance colonoscopy was performed after 2 years of Tocilizumab therapy. Normal mucosa was seen from rectum to terminal ileum. Histology of random biopsies showed no active inflammation. There was chronic inflammation and crypt distortion in the rectum, but it was normal in colon and terminal ileum. The appearances were consistent with quiescent colitis.

No major adverse event was detected. She reported 6 episodes of respiratory tract infections over the 24-month period. Each episode was successfully managed by her primary care physician with oral antibiotics. At her 12-month follow-up, she was suffering from an episode of respiratory tract infection which precipitated a flare-up of RA. This resolved after treatment of the infection.

## 3. Discussion

We found 1 other case of Tocilizumab therapy in a patient with UC. In contrast to our report, it reported failure of Tocilizumab therapy in a UC patient. The authors reported endomicroscopic and histological evidence of worsened inflammation after 12 weeks of Tocilizumab therapy. It should be noted that azathioprine was stopped in that patient when Tocilizumab was started [6]. It is uncertain whether the exacerbation was due to azathioprine withdrawal or introduction of Tocilizumab.

Our literature search found evidence for the involvement of the IL6 pathway in development and maintenance of inflammation in UC. Elevated levels of IL6 are found in bowel mucosa and serum of UC patients [2]. Use of anti-IL6 receptor monoclonal antibody has been shown to reduce inflammation in a murine colitis model [1].

We feel that further controlled clinical trials to evaluate the therapeutic value of IL6 pathway inhibition in UC are warranted. Further studies into the histological, immunological, and genetic characteristics of responders and nonresponders can also enhance understanding of underlying pathophysiology.

In clinical trials, the rate of gastrointestinal perforation was 1.9 per 1000 patient-years in Tocilizumab arms, compared to 1.3 per 1000 patient-years in control arms [5]. The manufacturer therefore cautioned against the use of Tocilizumab in patient with gastrointestinal ulcerations.

In practice, RA patients without effective DMARDs will be on glucocorticoid and/or nonsteroidal anti-inflammatory drugs (NSAIDs). These are strong risk factors for gastrointestinal perforation in RA patients [7]. In our case, commencing Tocilizumab therapy has enabled the patient to discontinue prednisolone. Therefore we feel that Tocilizumab is not absolutely contraindicated in patients with gastrointestinal ulceration, and clinicians should consider the risk and benefits in each individual patient.

Further studies to define the risk of Tocilizumab-related gastrointestinal toxicity in “real-life” setting are indicated to inform treatment decisions in RA patients with coexisting gastrointestinal disease. Cohort study using registry data can help define risk of gastrointestinal perforation associated with biologic DMARDs [8]. Many countries have already set up national registers of RA patients treated with Tocilizumab [9], enabling the relevant study to be performed in the near future.

This is the first case report of successful Tocilizumab use in patient with coexisting UC and RA. The clinical responses in both conditions were sustained over two years. Tocilizumab is a potential therapeutic option in UC and further controlled study is warranted. Tocilizumab related gastrointestinal toxicity needs to be balanced against that of glucocorticoid and NSAIDs therapy. Analysis of registry data will allow better understanding of such risk.

## Figures and Tables

**Table 1:** Summary of medical treatment offered to patient.

	Duration	Reason for discontinuation
*Drug for RA*		
Methotrexate (5 mg per week)	Max 15 months^*∗*^	Nausea
Methotrexate (7.5 mg to 10 mg per week)	Max 10 months^*∗*^	Nausea, malaise
Sulfasalazine	Less than 3 months	Mouth ulcers
Hydroxychloroquine	6 months	Taste disturbance
Leflunomide	30 months	Mouth and oesophageal ulcers
Azathioprine	3 months	Nausea, headache
Etanercept (monotherapy)	2 months	Facial flush, panic attacks
Infliximab (in combination with Methotrexate)	11 months	Facial flush, headache, depression
*Drug for UC*		
Asacol MR	4 months	Oral and pharyngeal mucositis
*Treatments declined by patient*		
Ciclosporin	Worried about side effects	
Other oral mesalazine preparations		
Rituximab		

^*∗*^Methotrexate was rechallenged alone or in combination on multiple occasions.

**Table 2:** Disease activity of RA at baseline and follow-up period.

	Baseline	3 months	6 months	12 months	18 months	24 months
Tender joints (0–28)	24	2	2	10	2	1
Swollen joints (0–28)	10	4	0	6	0	0
ESR (mm/h)	52	3	7	8	7	11
Global Health score (0–100)	80	50	40	60	40	60
DAS28 score	7.81	2.82	2.71	4.75	2.71	3.08
Prednisolone dose (mg/day)	10	2.5	2.5	2.5	0	0

**Table 3:** Laboratory inflammatory markers at baseline and follow-up period.

	Reference range	Baseline	3 months	6 months	12 months	18 months	24 months
Haemoglobin (g/L)	110–150	**103**	**101**	110	**107**	113	110
White blood cells (10^9^/L)	4–11	10.8	4.9	6.3	5.2	7.1	5.7
Platelets (10^9^/L)	150–400	**553**	374	**442**	**439**	**422**	**421**
Mean cell volume (fL)	80–100	**75.3**	81	85.5	81.7	82.8	83.8
ESR (mm/h)	1–20	**52**	3	7	8	7	11
CRP (mg/L)	0–10	2	<1	<1	<1	<1	<1
Albumin (g/L)	35–50	39	38	40	41	39	41
Weight (kg)	—	87	92	94	96	96	96

ESR: Erythrocyte Sedimentation Rate, CRP: C-Reactive Protein.
